# Long-term follow-up results in patients with thoracolumbar unstable burst fracture treated with temporary posterior instrumentation without fusion and implant removal surgery: Follow-up results for at least 10 years

**DOI:** 10.1097/MD.0000000000019780

**Published:** 2020-04-17

**Authors:** Sangbong Ko, Sukhan Jung, Sukkyoon Song, Jun-Young Kim, Jaibum Kwon

**Affiliations:** Daegu Catholic University Medical Center, Daegu, South Korea.

**Keywords:** fracture, fracture fixation, spinal

## Abstract

Segmental fusion is not necessarily needed in treatment of thoracolumbar unstable burst fracture requiring surgery. Our objective was to report the results of follow-up for at least 10 years in patients with thoracolumbar unstable burst fracture requiring surgery in which fractured segment was healed following temporary posterior instrumentation without fusion, and in whom implants were subsequently removed.

Retrospective Cohort Study.

Nineteen patients in whom union of fractured vertebra was observed following surgery and in whom implants were removed within an average 12.2 months, and who could be followed up for at least 10 years, were enrolled.

At the last follow-up, we evaluated the segmental motions, anterior body height ratio, progress of further kyphotic deformity, Oswestry Disability Index, Rolland Morris Disability Questionnaire and Short Form 36.

Results: The follow-up period after implant removal surgery was 151 months on average. The local kyphotic angle was 26.89 ± 6.08 degrees at the time of injury and 10.11 ± 2.22 degrees at the last follow-up. The anterior body height ratio was 0.54 ± 0.16 at the time of injury and 0.89 ± 0.05 at the last follow-up. Thus, the fractured vertebra was significantly reduced after surgery and maintained till last follow-up. The segmental motion was 9.84 ± 3.03, Oswestry Disability Index was 7.95 ± 7.38, Rolland Morris Disability Questionnaire was 2.17 ± 2.67, short form 36 Physical Component Score was 77.50 ± 16.61, and short form 36 Mental Component Score was 79.21 ± 13.32 at last follow-up.

We conducted at least 10-year follow-up and found that temporary posterior instrumentation without fusion should be considered one of the useful alternative treatments for thoracolumbar unstable burst fracture in place of the traditional posterior instrumentation and fusion.

## 1 Introduction

Thoracolumbar unstable burst fracture is one of the most common spine factures, requires surgery. There may be various surgical options on the proper treatment modality, from simple non-surgical treatment to complicated posterior-anterior-posterior approach considering the postoperative stability of the fracture fragment. However, there is consensus that acute, unstable, severe kyphotic deformity or those with damage to the middle osteoligamentous complex and posterior ligament complex require surgical treatment in most cases.^[1–10]^ Of numerous surgical treatment options available, anterior or posterior instrumentation with segmental fusion is frequently used for the stability of a fractured spine,^[11–14]^ although temporary posterior instrumentation without fusion is also used.^[15,16]^

In the case of thoracolumbar unstable burst fracture requiring surgery, temporary posterior instrumentation without fusion has a number of strong points. That is, it can align fractured spines and retain vertebral height and canal dimension by repositioning posteriorly displaced bone fragments through indirect reduction by ligamentotaxis, as well as is less invasive. And, this technique prevent many problems caused by fusion of mobile segment by preserving motion segments even after union of fractured vertebra and subsequent implant removal.^[17]^ However, it has also weak points, including early fixation failure by insufficient anterior support with only posterior fixation.^[18–20]^ McCormack et al^[19]^ have tried to address this weakness and contended that anterior support is required for patients with a load-sharing score of seven points or more, since posterior fixation alone was not enough to ensure stability. Besides early fixation failure, reduction loss in the intervertebral disc following implant removal has also been reported recently.^[21]^ Although there are several strong and weak points, some spine surgeons have suggested that temporary posterior instrumentation without segmental fusion may replace conventional posterior instrumentation with segmental fusion.^[21–25]^ However, they evaluated only the stability of the fracture site by short segment fixation and the functional outcome for not-long period. In addition, there were little researches on whether segmental motion is lost in long-term follow-up after removal of implant, and whether additional kyphotic deformity does not progress over time.

The purpose of this study was to evaluate the outcome of at least 10 years follow-up after union of the fractured spine by posterior instrumentation without fusion in thoracolumbar unstable burst fractures requiring surgery, to confirm whether mobile segment was preserved, and whether functional outcomes or quality of life deteriorated. This is the first report of a long-term follow up of more than 10 years using this non-fusion technique.

## 2 Methods

### 2.1 Patient populations

Patients with thoracolumbar unstable burst fracture requiring surgery owing to definite posterior ligament injuries detected with magnetic resonance imaging (MRI) or computed tomography (CT) before surgery, and who could be followed up for at least 10 years after healing of fractured segments and removal of implants were enrolled. This study excluded patients who had severe neurological deficit requiring anterior decompression, who were child or aged 50 years or older at surgery, who were unable to tolerate surgical treatment because of other severe medical illness, who had burst fracture of two segments or more, who could not receive primary instrumentation within 1 week after injury, and those who had secondary gains such as worker's compensation. We also excluded patients who had a McCormack load-sharing score of seven points or more requiring additional anterior support. McCormack load sharing classification score is composed of comminution(3 points, Little(30% comminution on sagittal plane CT) = 1, More(30%–60%) = 2, Gross(>60%) = 3), apposition of fragments (3 points, Minimal = 1, Spread (2 mm displacement of <50% cross-section of body) = 2, Wide(>50%) = 3) and reducibility of sagittal deformation(3 points, Little (Kyphotic correction ≤3°) = 1, More (4°∼9°) = 2, Most (≥10°) = 3). Severely comminuted fractures scoring 7 points or more supposed to be repaired by an anterior approach with partial corpectomy and strut grafting and excluded in this study.

From March 1, 2004 to January 31, 2007, total 27 patients who met the inclusion and exclusion criteria were eligible. In one case, at the 2-year follow-up, nonunion of the fractured vertebral body was found by an intervertebral disc inserted in the fracture vertebral body, which additional posterior segmental fusion surgery was required. In one case, implant removal surgery was rejected due to spontaneously anterior segmental fusion. Four cases had follow-up loss (2 cases moved to another city and 2 cases were telephone and address unknown). One patient died of another problem such as pancreatic cancer. One patient refused to complete the questionnaire and check the X-ray due to death of spouse. Thus, 19 patients out of a total 27 patients were enrolled.

### 2.2 Management

**Figure 1:**
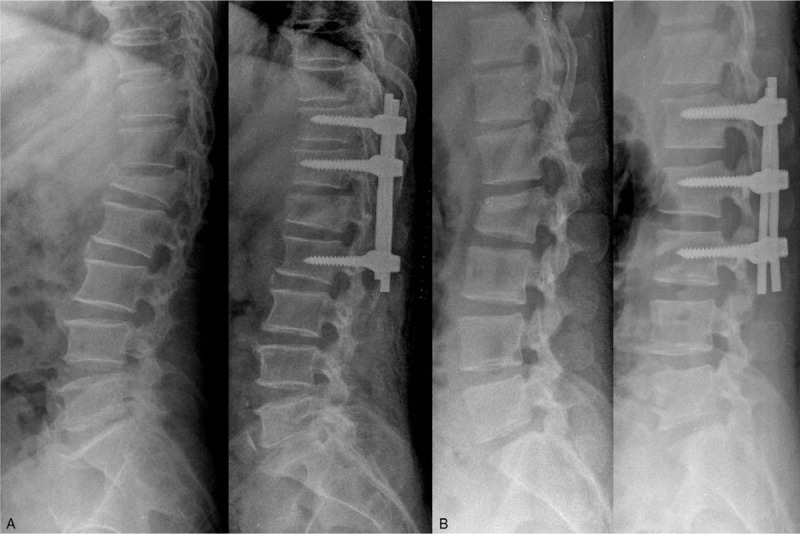
(A) Basically instrumented the upper 2 levels and lower 1 level in the fracture site using pedicle screws. (B) If the pedicle of the fractured vertebra is appropriate for pedicle screw insertion, 1 mono-axial pedicle screw was inserted to the fractured vertebra and short segment fixation was performed from 1 level above to 1 level below the fracture vertebra.

Posterior instrumentation without fusion was performed within an average of 2.8 days (range: 0–6 days) after injury. The midline skin is incised and bilateral fascia is incised separately. We used a paramedian approach accessing the posterolateral aspect of the spine through a passage between the multifidus and longissimus muscles using finger dissection for minimum invasion to nearby soft tissues. We basically instrumented the upper 2 levels and lower one level in the fracture site using pedicle screws (Fig. 1A). If the pedicle of the fractured vertebra is appropriate for pedicle screw insertion, 1 mono-axial pedicle screw was inserted to the fractured vertebra and short segment fixation was performed from 1 level above to 1 level below the fracture vertebra (Fig. 1B). Following pedicle screw-rod fixation, posteriorly displaced bone fragments were reduced by the distraction of each level such as ligamentotaxis. To avoid violation to the facet joint, convergent placement of pedicle screws was recommended from the lateral through the medial aspect of facet joints. Laminotomy or laminectomy, as well as additional procedures such as bone graft for superior-inferior lumbar interbody fusion in the injured site or decortication were not conducted. Patients started to ambulate the day after surgery wearing Thoraco-Lumbo-Sacral Orthosis (TLSO) Patients treated with 2 level above 1 level below fixation wore TLSO for 6 weeks, patients treated with 1 level above, 1 level below fixation wore TLSO for 3 months. No patients wore TLSO longer than 3 months. Implant removal surgery was conducted to remove pedicle screws 12.2 months (range: 8–15 months) following primary surgery using an existing paramedian inter-muscular approach.

### 2.3 Radiographic and functional outcome evaluation

**Figure 2:**
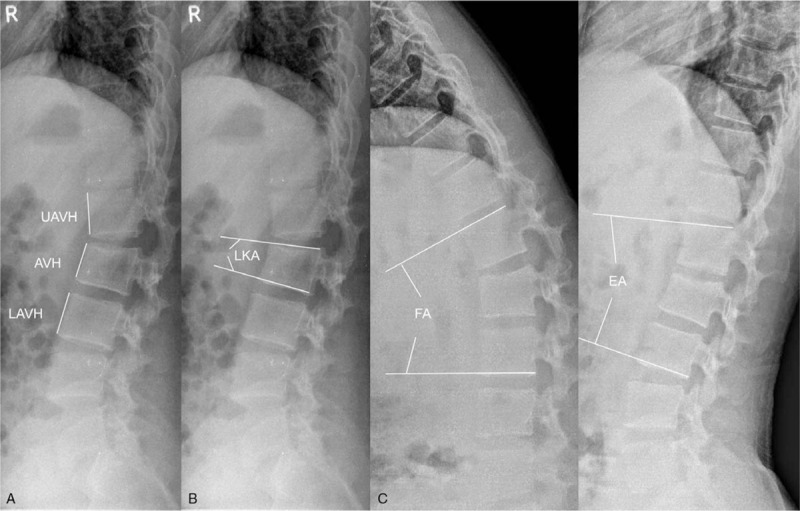
Plane radiographs of an 18-year-old man with an L2 burst fracture. Standing lateral image after removal surgery showing the (A) ABHR and (B) LKA; ABHR = AVH/(UAVH + LAVH)/2, LKA: the angle between the superior and inferior end plate of the fractured vertebra. (C) By 10 years after removal surgery, we determined the inter-segmental motion angle (flexion angle subtracted from the extension angle) by Cobb angle method. AVH = anterior vertebral body height of the fractured vertebra, LAVH = lower anterior vertebral body height, UAVH = upper anterior vertebral body height, FA = flexion angle, EA = extension angle.

At the time of injury, we took images of subjects including plain radiographs, MRI, and CT scans in the emergency room. On the basis of preoperative imaging study, we classified all fractures using AO Spine Classification System and scored using McCormack load-sharing score system. If there is union in the fracture site, standing plain radiographs, and CT scans were taken prior to implant removal surgery. Specifically, the absence of motion in the fracture site was identified on flexion-extension standing radiographs using Cobb method. From CT scans, we reconfirmed the union of fractured vertebral body, and evaluated the severity of facet joint arthritis.^[26]^ The facet joints were decided to become worse if four joints adjacent of fractured vertebra progressed more than twice as compared preoperative CT finding. After removal surgery, plain radiography consisting of standing antero-posterior images, standing lateral image, and standing flexion-extension dynamic image was annually carried out in order to measure anterior body height ratio (ABHR) (Fig. 2A) and local kyphotic angle (LKA) (Fig. 2B). At least 10 year follow up following removal surgery, we determined the inter-segmental motion angle (flexion angle subtracted from the extension angle) by measuring the angle between the superior endplate of the superior segment and the inferior endplate of the inferior segment in the fracture site with reference to flexion-extension lateral standing radiographs, by Cobb angle method (Fig. 2C).

In order to evaluate functional outcomes related to the spine in all patients, the Oswestry Disability Index (ODI) and Rolland Morris Disability Questionnaire were used. To evaluate quality of life in all patients, short form 36 (SF-36) Physical Component Score (PCS) and SF-36 Mental Component Score (MCS) were measured. The functional outcomes and quality of life were assessed 3 times before removal, 1 year, and at least 10 years after removal.

### 2.4 Statistical analysis

All statistical analyses were performed using a paired t-test in SPSS 18.0 (SPSS Inc., Chicago, IL). The result of functional outcome and quality of life were analyzed using a repeat measure one factor analysis. Post-hoc test was analyzed by multiple comparison result by contrast. A *P* value of less than .05 was considered statistically significant.

### 2.5 Ethical statement

This study was approved by Daegu Catholic Medical Center IRB. Approval number is CR-14–055. Waiver of Documentation of Consent was confirmed by IRB, because, this study is retrospective study based on medical record and data obtained was protected in secured storage. This study also has no possibilities to benefit or harm patients who involved in.

## 3 Results

### 3.1 Demographical characteristics

Patients enrolled for this study consisted of 8 males and 11 females. The mean age was 34.8 years (range: 18–49 years). The mean duration to implant removal surgery following primary surgery was 12.2 months (range: 8–15 months). The mean follow-up duration in implant removal surgery was 151 months (range: 120–168). Fracture sites included T11 in 1 case (5.26%), T12 in 4 cases (21.05%), L1 in 8 cases (42.11%), L2 in 5 cases (26.32%), and L3 in 1 case (5.26%). Causes of injury were traffic accident in 4 cases (21.05%), fall from height in 12 cases (63.16%), and direct injury in 3 cases (15.79%). AO classification by preoperative imaging study was 6 cases in B2, 8 cases in A3, and 5 cases in A4. According McCormack load sharing classification score system, score 3 was 2 case, score 4 was 3 cases, score 5 were 6 cases, and score 6 was 8 cases. Demographic characteristics of all patients were listed in Table 1.

**Table 1:**
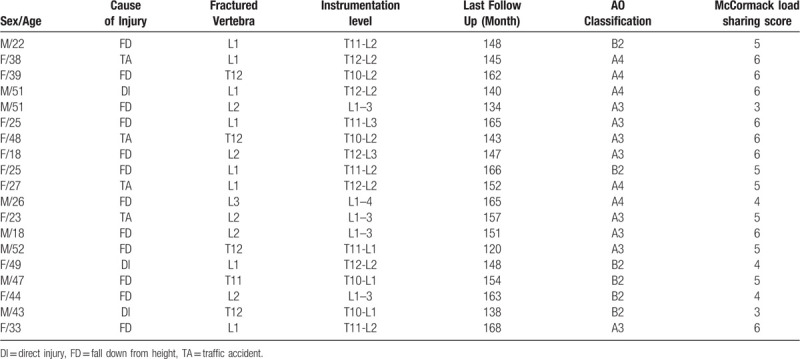
Demographical characteristics of all patients.

### 3.2 Radiological results

**Figure 3:**
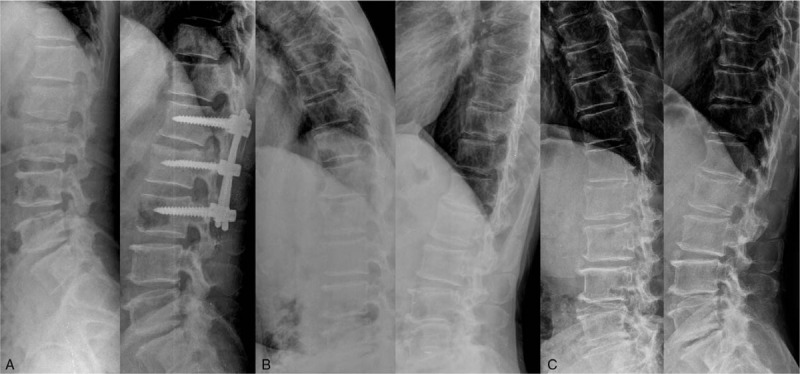
Lateral radiographs of a 51-year-old man with L1 burst fracture. Preoperative and immediate postoperative radiographs (A), flexion-extension lateral standing radiographs at 15 months (B), and 140 months after implant removal surgery (C). The segmental motion measured from flexion-extension lateral standing radiographs were 7.52 degrees (B) and 6.29 degrees (C), respectively.

Average LKA was 26.89 ± 6.08 (range: 17–45) degrees at the time of injury, 10.37 ± 1.98 (range: 7–14) degrees at one year after implant removal surgery, and 10.11 ± 2.22 (range: 6–14) degrees at the last follow-up, showing significantly reduction state compared to that at the time of injury (*P* < .05). However, there was no significant different in LKA value between 1 year after the removal surgery and the last follow-up (*P* = .71). Average ABHR was 0.54 ± 0.16 at the time of injury, 0.89 ± 0.04 at 1 year after the removal surgery, and 0.89 ± 0.05 at the last follow-up. These results confirmed that fracture vertebra was significantly repositioned compared to that at the time of injury (*P* < .05). However, there was no significant difference in ABHR value between 1 year after the removal surgery and the last follow-up (*P* = .87). The segmental motion was measured to be 10.43 ± 3.32 (range: 1–16) degrees at the 1 year after removal surgery, 9.27 ± 3.34 (range: 3–16) degrees at the last follow up after removal surgery, on average, respectively, showing that it was statistically significantly decreased with the passage of time (*P* = .028) (Fig. 3). In the severity of facet joint injury by preoperative CT findings, 17 patients were Grade 1 and 2 patients were Grade 2. By pre-removal CT findings, 16 patients were Grade 1, 2 patients were Grade 2, and only 1 patient was Grade 3. Radiological results of all patients were listed in Table 2.

**Table 2:**
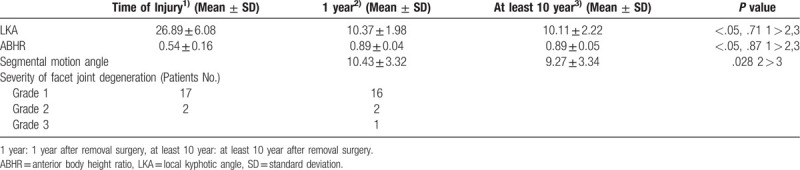
Radiological results of all patients.

### 3.3 Results of functional outcome and quality of life

ODI was 15.86 ± 7.93 at the time of removal surgery, 10.60 ± 5.67 one year after implant removal surgery, 7.95 ± 7.38 at the last follow-up, respectively, showing statistically significant improvement over time (*P* < .001). Rolland Morris Disability Questionnaire was 7.23 ± 4.47 at the time of removal surgery, 4.83 ± 4.18 one year after implant removal surgery, 2.17 ± 2.67 at the last follow up, respectively, showing statistically significant improvement over time (*P* < .001). In addition, for the evaluation of quality of life the SF-36 PCS was 40.71 ± 18.67 at the time of removal surgery, 56.58 ± 21.56 one year after implant removal surgery, 76.73 ± 17.24 at the last follow up, respectively, showing statistically significant improvement over time (*P* < .001). SF-36 MCS was 50.24 ± 21.32 at the time of removal surgery, 62.08 ± 20.31 one year after implant removal surgery, 78.58 ± 13.27 at the last follow up, respectively, showing that SF-36 MCS was statistically significantly improved over time (*P* < .001) (Table 3).

**Table 3:**
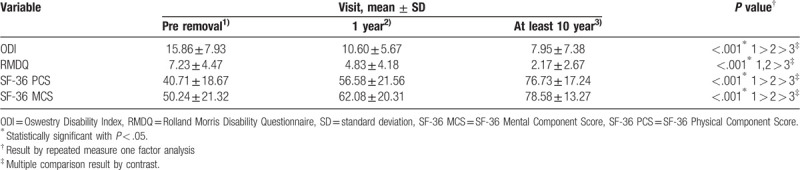
Results of functional outcome and quality of life by visit.

## 4 Discussions

In terms of short-segmental pedicle screw fixation without anterior support as a treatment for thoracolumbar unstable burst fracture, there are several problems besides early instrument fixation failure or progressive kyphotic deformity.^[18–20]^ Specifically, Aono et al^[21]^ have contended that if titanium with strength and elasticity 2 times greater than the conventional stainless steel^[27,28]^ is used as screw materials, the risk of early instrument fixation failure is decreased because the material used can modify the risk of failure. Another study has reported that using pedicle screws with as thick a diameter as possible can also prevent failure.^[29]^

For recurrent kyphotic deformity, Aono et al^[21]^ have contended that temporary fixation alone is enough to reposition and maintain fracture sites, although the degree of bone fragment displacement into spinal cavity or the degree of kyphotic angle is one of factors contributing to kyphotic deformity after implant removal surgery. Some authors have also reported that the progress of kyphotic deformity can be prevented by additional load-sharing provided by additionally conducted vertebroplasty.^[30,31]^ However, based on MR images taken 2 years after surgery, Choi et al^[32]^ have reported that disc degeneration could be deteriorated only by inter-segmental immobilization and could be greatly aggravated through endplate injury due to fractures. The results of Choi et al^[32]^ have demonstrated that post-removal kyphotic deformity developing after implant removal surgery, which have been described by Aono et al,^[21]^ was attributable to the loss of disc height due to disc degeneration rather than the vertebral body itself. Consistent with Spiegl et al,^[33]^ we did not conduct MRI again to confirm disc status during the long-term follow-up despite its importance. However, in 2014, Ko et al^[17]^ reported that LKA was decreased by approximately 1.22 ± 0.60 degrees at 2 to 7 years after the removal surgery although such decrease was not statistically significant. In the present study, long-term follow-up results showed that LKA loss was present at an early stage after the removal surgery. It did not continue to advance. In a 2-year study, Aono et al^[21]^ had pointed out some degree of kyphosis progress. However, in our study, no further progress of LKA was observed during the follow-up time of at least 10 years.

There may be a problem with the integrity of facet joints owing to long-term immobilization. Tromme et al^[34]^ have reported that degenerative osteoarthritis or the spontaneous fusion of facet joints can occur by long-term immobilization. Specifically, degenerative osteoarthritis is deteriorated mainly by cement augmented screw insertion with a frequency of 7.5%. Spontaneous fusion occurs in the anterior part of facet joints in the case of B type injuries (according to AO classification). It occurs more frequently in some conditions, including old age, high body mass index, degenerative alteration in the facet joint before surgery, and a longer period from fixation to implant removal surgery, with an overall frequency of 3.6%. Similarly, in the present study, spontaneous fusion occurred in one case of B type fractures (according to AO classification). Pedicle screws were inserted via a convergent path from the lateral to the medial aspects of facet joints to minimize the risk of facet joint violation through the paramedian approach. These joints were immobilized in just 12 months after fixation. For flexion-extension movement, there were no problems caused by the level of preservation for degenerative osteoarthritis of facet joints, since subjects were relatively young (mean age of 35 years) at the time of fixation surgery. At the last follow-up, there were pain-free joint motions with an angle of approximately 9.84 ± 3.03 degrees. Facet joint deterioration was observed by Tromme et al^[34]^ during average follow-up period of 12.3 months. In our study, although the exact degeneration may be not easy to assess due to lack of further CT scans, there were no decrease in joint motion range and patients did not complain of pain during motion. On this basis, degenerative changes are thought to progress no further. However, additional studies are thought to be needed about this subject.

Of spine-related functional outcomes measured at least 10 years after the removal surgery, ODI was 7.95 ± 7.38. This represents minimal disability (scores from 0% to 20%) based on a classification proposed by Fairbank et al.^[35]^ In addition, for the evaluation of quality of life in general patients, SF-36 PCS and MCS were 77.50 ± 16.61 and 79.21 ± 13.32, respectively. Both indices confirmed that they had excellent quality of life corresponding to nearly the top 80% on a normal distribution. However, SF-36 is not enough to evaluate quality of life because it represents relative values, not absolute values. Notably, we had trouble making a comparative study with other studies due to the absence reporting functional outcomes measured at least 10 years after implant removal or functional outcomes of the fusion group due to other factors that should be considered such as surgical method, long-term follow-up results, instability of adjacent segments related to fusion, and disc degeneration of adjacent segments.

This study has some limitations. First, results of temporary posterior instrumentation without fusion should be compared with those of posterior instrumentation with fusion in studies for patients with thoracolumbar unstable burst fracture. However, there was no control group for this comparison. In the future, more specific results should be collected in a randomized comparison study for the 2 surgeries. Second, at the last follow-up, we measured spinal alignment based on plain radiographs while, joint motion and progress of additional kyphotic deformity were based on flexion-extension images. Additional MRI or CT scans were not taken to examine more clear results for facet joint integrity or disc degeneration. Third, more patients are required for more accurate analysis. In fact, based on inclusion/exclusion criteria, patients with the following conditions were considered ineligible for this study. Fourth, in nonfusion surgery, screw loosening may be frequent. In this study evaluation of screw loosening was not performed but definite screw loosening or rod breakage was not noticed during removal surgeries. Fifth, work ability or occupation change due to disability was not checked during follow-up.

## 5 Conclusion

In patients with thoracolumbar unstable burst fracture who had a McCormack load-sharing score of less than 7 points, injured posterior ligaments, and operation indication, we found that the fracture site was healed after temporary posterior instrumentation without fusion and implants were subsequently removed. As a result of at least 10 years of follow-up, there were no additional crucial local kyphotic alterations or reduction loss of the fracture site. In addition, the joint motion range was maintained at an angle of nearly 10 degrees even after implant removal without adjacent segment complications. Furthermore, patients’ functional outcome and quality of life improved over time and showed minimal disability at last follow-up, with all patients being in the 80th percentile. Therefore, among the alternative treatments of posterior instrumentation with segmental fusion, temporary posterior instrumentation without segmental fusion should be considered as an excellent treatment option for patients at least 10 years after removal surgery.

## Author contributions

**Conceptualization:** Sangbong Ko.

**Data curation:** Sangbong Ko, Sukhan Jung, Jun-Young Kim.

**Formal analysis:** Sukkyoon Song.

**Investigation:** Sangbong Ko, Sukhan Jung.

**Methodology:** Sangbong Ko, Sukhan Jung, Sukkyoon Song.

**Supervision:** Sangbong Ko, Sukhan Jung, Jaibum Kwon.

**Validation:** Sangbong Ko, Sukhan Jung, Sukkyoon Song, Jaibum Kwon, Jun-Young Kim.

**Writing – original draft:** Sangbong Ko.

**Writing – review & editing:** Sukhan Jung, Sukkyoon Song, Jaibum Kwon, Jun-Young Kim.

Sukhan Jung orcid: 0000-0001-8966-1486.
